# P14 Multidisciplinary antimicrobial stewardship: transforming COVID-19 pandemic response and combating global antimicrobial resistance in UK healthcare settings

**DOI:** 10.1093/jacamr/dlaf230.021

**Published:** 2025-12-04

**Authors:** Rasha Abdelsalam Elshenawy, Nkiruka Umaru, Zoe Aslanpour

**Affiliations:** Department of Medicine, School of Health, Medicine and Life Sciences, University of Hertfordshire, Hatfield, UK; Department of Medicine, School of Health, Medicine and Life Sciences, University of Hertfordshire, Hatfield, UK; Department of Medicine, School of Health, Medicine and Life Sciences, University of Hertfordshire, Hatfield, UK

## Abstract

**Background:**

Multidisciplinary collaboration emerges as the cornerstone of effective antimicrobial resistance (AMR) prevention in modern healthcare. With MDR infections threatening to claim 10 million lives annually by 2050, the urgency for integrated healthcare solutions has never been greater. Sir Alexander Fleming's early warnings about AMR now resonate powerfully as the COVID-19 pandemic has accelerated the need for robust antimicrobial stewardship (AMS) programmes that unite doctors, pharmacists and nurses in a coordinated fight against superbugs.^1^ This research reveals how pandemic-driven healthcare transformations can strengthen our defence against one of medicine's most significant challenges.^2,3^

**Objectives:**

To investigate how multidisciplinary antimicrobial stewardship practices evolved during COVID-19, examining healthcare professionals' collaborative approaches to antibiotic prescribing, resistance prevention and patient safety optimization across pre-pandemic and pandemic periods at an NHS Foundation Trust in the East of England.

**Methods:**

This comprehensive three-study investigation employed mixed-methods approaches to capture the full spectrum of antimicrobial stewardship evolution. Study 1 systematically reviewed global AMS implementation strategies using rigorous postpositivist methodology. Study 2 retrospectively analysed 640 patient records across pre-pandemic and pandemic periods, utilizing SPSS statistical analysis to identify prescribing pattern changes. Study 3 prospectively surveyed 240 healthcare professionals (doctors, pharmacists, nurses) using validated questionnaires, employing descriptive statistics and regression analysis to quantify attitude shifts and practice modifications during the pandemic.

**Results:**

Revolutionary findings emerged across all studies. Study 1 demonstrated that 92% of successful AMS programmes featured multidisciplinary teams integrating infectious disease physicians, clinical microbiologists and pharmacists, with effective communication proving crucial for implementation success. Study 2 revealed enhanced collaborative care during the pandemic, with pharmacist involvement in antimicrobial decisions increasing from 19% to 21%, signalling strengthened interdisciplinary cooperation. Study 3 uncovered significant awareness shifts, with 42% of professionals acknowledging COVID-19's amplification of antimicrobial resistance risks, while 60% of pharmacists reported increased enthusiasm for AMS activities. However, compliance with local antimicrobial guidelines remained challenging at approximately 22%, highlighting implementation gaps requiring targeted AMS intervention.

**Conclusions:**

Multidisciplinary antimicrobial stewardship represents the future of infection control and resistance prevention in healthcare. This research provides compelling evidence that collaborative approaches between doctors, pharmacists and nurses are not merely beneficial but essential for combating AMR, particularly during health crises like COVID-19. The pandemic has catalysed unprecedented cooperation among healthcare professionals, creating opportunities to strengthen antimicrobial stewardship programmes through enhanced communication, shared decision-making and integrated care pathways. These findings offer actionable insights for healthcare organizations worldwide seeking to optimize their antimicrobial use policies and protect patients from the growing threat of drug-resistant infections.
